# Generation of Transgenic Porcine Fibroblast Cell Lines Using Nanomagnetic Gene Delivery Vectors

**DOI:** 10.1007/s12033-016-9934-1

**Published:** 2016-04-05

**Authors:** Bartosz F. Grześkowiak, Magdalena Hryhorowicz, Karol Tuśnio, Mikołaj Grzeszkowiak, Karol Załęski, Daniel Lipiński, Joanna Zeyland, Olga Mykhaylyk, Ryszard Słomski, Stefan Jurga, Anna Woźniak

**Affiliations:** The NanoBioMedical Centre, Adam Mickiewicz University, Umultowska 85, 61-614 Poznan, Poland; Department of Biochemistry and Biotechnology, Poznań University of Life Sciences, Dojazd 11, 60-632 Poznan, Poland; Institute of Molecular Immunology and Experimental Oncology, Technical University Munich, Ismaninger Str. 22, 81675 Munich, Germany; Department of Macromolecular Physics, Faculty of Physics, Adam Mickiewicz University, Umultowska 85, 61-614 Poznan, Poland

**Keywords:** Nucleic acid delivery, Magnetofection, Magnetic nanoparticles, Transgenesis, Porcine fetal fibroblasts

## Abstract

The transgenic process allows for obtaining genetically modified animals for divers biomedical applications. A number of transgenic animals for xenotransplantation have been generated with the somatic cell nuclear transfer (SCNT) method. Thereby, efficient nucleic acid delivery to donor cells such as fibroblasts is of particular importance. The objective of this study was to establish stable transgene expressing porcine fetal fibroblast cell lines using magnetic nanoparticle-based gene delivery vectors under a gradient magnetic field. Magnetic transfection complexes prepared by self-assembly of suitable magnetic nanoparticles, plasmid DNA, and an enhancer under an inhomogeneous magnetic field enabled the rapid and efficient delivery of a gene construct (pCD59-GFPBsd) into porcine fetal fibroblasts. The applied vector dose was magnetically sedimented on the cell surface within 30 min as visualized by fluorescence microscopy. The PCR and RT-PCR analysis confirmed not only the presence but also the expression of transgene in all magnetofected transgenic fibroblast cell lines which survived antibiotic selection. The cells were characterized by high survival rates and proliferative activities as well as correct chromosome number. The developed nanomagnetic gene delivery formulation proved to be an effective tool for the production of genetically engineered fibroblasts and may be used in future in SCNT techniques for breeding new transgenic animals for the purpose of xenotransplantation.

**Electronic supplementary material:**

The online version of this article (doi:10.1007/s12033-016-9934-1) contains supplementary material, which is available to authorized users.

## Introduction

Since the term “transgenic” was used for the first time [1], there has been a growth in the application of genetically modified animals in diverse biological and medical areas, both for scientific and economic purposes [2–5]. Somatic cell nuclear transfer (SCNT), known as cloning, holds the greatest promise for significant improvements in the creation of transgenic animals. This method enables cell manipulation in vitro including the addition of a suitable gene or inactivation of an endogenous gene as well as selection of modified cells and their storage for future application. It can result in developing cheaper and easier procedures for transgenic animal production [6]. In the SCNT technique, efficient nucleic acid delivery into somatic cells such as fibroblasts is of particular importance. A wide range of biological, chemical, and physical methods for improving gene transfection have been applied in SCNT [7–10]. Despite high transduction efficiency and long-term gene expression, viral vectors suffer from numerous shortcomings including immunogenicity, insertional mutagenesis risk, expensive large-scale production, and limited packaging capabilities [11]. Compared to viral vectors, synthetic non-viral carriers possess many advantages such as reduced risk of immune response, low production cost, ease of modification, and better storage stability. However, the non-viral vectors have unsatisfactory transfection efficiency in many cell types [12]. Physical methods such as electroporation (nucleofection), employing physical force to facilitate intracellular gene transfer, exhibit a high transfection efficiency in various cell types but are limited by poor cell viability [13]. The improvement of the transgenic process can be achieved by developing a new, efficient, and safe system of transgene delivery into cells.

In non-viral gene delivery, inorganic nanoparticles such as carbon nanotubes, gold nanoparticles, quantum dots, and magnetic nanoparticles have received increasing attention due to their unique properties including small particle size, large surface area, surface modification possibility, stability, and biocompatibility [14]. A great advantage of magnetic nanoparticles is their magnetic responsiveness in external magnetic fields. Magnetofection is defined as nucleic acid delivery into cells under the influence of a magnetic field acting on nucleic acid vectors that are associated with magnetic nanoparticles [15]. This physical method uses magnetic force to sediment the magnetic vectors onto the surface of the cells to be transfected [16, 17]. The viral vectors, lipoplexes, and polyplexes are usually non-aggregated nanoparticles. Therefore, they are able to reach target cells in culture mainly via diffusion. Diffusion is a slow process and many vector types are prone to time-dependent inactivation under cell culture conditions, and are also toxic to cells in high concentrations. Thus, the use of magnetofection can greatly improve transfection efficacy. The major advantages of magnetofection for gene delivery are its ability to overcome limitations to diffusion, improvement of the kinetics of the delivery process, and a significant reduction of applied vector doses for effective gene expression [18].

**Fig. 1:**
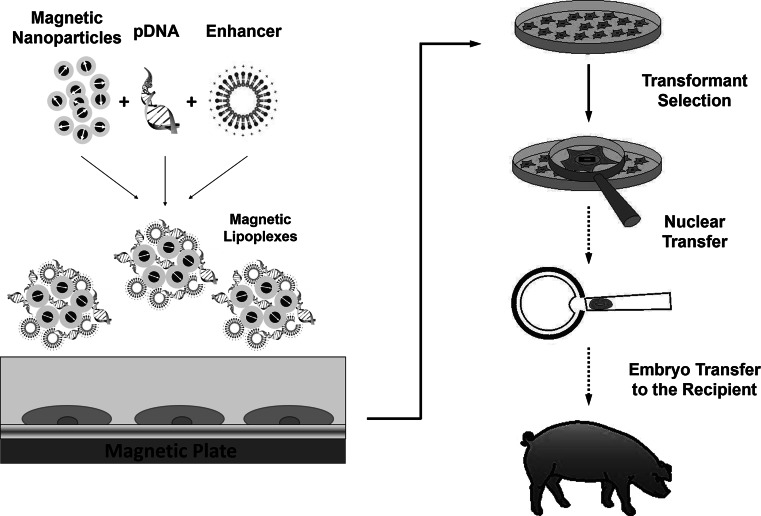
Schematic presentation of the magnetic nanoparticle-mediated generation of transgenic pigs. After formulation of magnetic complexes composed of PEI-Mag2 magnetic nanoparticles, a pCD59-GFPBsd gene construct containing human CD59 gene and DF-Gold as an enhancer, the complexes were associated with the porcine fibroblasts upon application of inhomogeneous magnetic field to promote transgene delivery. After 8-day antibiotic selection, verification of the transgene integration was performed to confirm the generation of transgenic cell lines, which could be further used for somatic cell nuclear transfer

In this study, magnetofection technology was used to deliver a pCD59-GFPBsd gene construct containing a gene encoding a protein involved in the regulation of the human immune response system, into porcine fetal fibroblasts (PFFs) (Fig. 1). These cells may be a source of nuclei in the SCNT method to generate transgenic animals for the purpose of xenotransplantation. PEI-Mag2 iron oxide nanoparticles with magnetic core and appropriate coating [18] were used to formulate efficient magnetic pDNA lipoplexes. To visualize the rapid internalization of magnetic complexes, images of fluorescently labeled pDNA were taken. To confirm integration of the transgene into the genome and its expression, PCR and RT-PCR analysis after 8-day selection was performed.

## Methods

### Cell Culture

Porcine fetal fibroblasts isolated from conceptuses, hereafter referred to as PFF cells, were cultured in Dulbecco’s modified Eagle’s medium (DMEM; Sigma-Aldrich, USA) supplemented with 10 % fetal bovine serum (FBS), 100 U/ml penicillin, 100 µg/ml streptomycin, and 0.25 µg/ml amphotericin B (Sigma-Aldrich). Every 3–4 days, the cells were split 1:2 at a seeding density of 1 × 10^4^ cells/cm^2^. Cells were cultivated on flasks coated with gelatin (30 min incubation with 0.1 % gelatin (Sigma-Aldrich) before cell seeding) at 37 °C in a humidified atmosphere containing 5 % CO_2_.

### Plasmid DNA

The pCD59-GFPBsd plasmid, which expresses the human CD59 gene under the control of the EF-1α promoter in the pTracer-EF/Bsd A (Invitrogen, USA) vector, was obtained, amplified, and purified as described elsewhere [19]. Luciferase reporter plasmid p55pCMV-IVS-luc+ containing the firefly luciferase cDNA under the control of the cytomegalovirus (CMV) promoter was amplified and purified by PlasmidFactory (Germany).

### Magnetic Nanoparticles for pDNA Delivery

Core/shell-type iron oxide magnetic nanoparticles (MNPs) for magnetic lipoplex formulation, hereafter referred to as PEI-Mag2, were synthesized by precipitating Fe(II)/Fe(III) hydroxide from an aqueous salt solution, followed by transformation into magnetite in an oxygen-free atmosphere, with immediate spontaneous adsorption of the 25-kDa branched polyethyleneimine (PEI-25_Br_) (Sigma-Aldrich) combined with the fluorinated surfactant ZONYL FSA (lithium 3-[2-(perfluoroalkyl)ethylthio]propionate) (Sigma-Aldrich), as described elsewhere [18, 20]. The physical size and morphology of the magnetic nanoparticle core was examined using high-resolution transmission electron microscopy (HR-TEM Jeol ARM 200F, Japan). To prepare TEM samples, a 10 µl drop of the nanoparticle suspension containing 10 µg iron was deposited onto a Lacey formvar/carbon-coated 200-mesh copper grid, incubated for 60 min, and dried under the vacuum of a 655 Turbo Pumping Station (Gatan, USA) prior to imaging at an accelerating voltage of 200 kV. The static magnetic properties of the particles in suspension were evaluated by magnetization measurement at 298 K using a SQUID magnetometer (MPMS-XL, Quantum Design, USA). The mean hydrodynamic diameter (*D*_*h*_) and electrokinetic potential (*ζ*) of the suspension of coated MNP in water were measured by photon correlation spectroscopy (PCS) using a Malvern Zetasizer Nano Series 3000 HS (Malvern Instruments GmbH, Germany). The morphologies of the MNPs were viewed using scanning electron microscopy (SEM, Jeol, JSM 7001F TTLS, Japan). A 10 µl drop of the nanoparticle suspension containing 10 µg iron was deposited onto a glass coverslip. The sample was dried in air and coated with chrome using a sputter coater/turbo evaporator (Quorum Technologies Q150T ES) for 45 s to provide a thin electrically conductive film to reduce thermal damage and charging of the samples. The images were taken at an accelerating voltage of 15 kV.

### Preparation and Characteristics of Transfection Complexes

For transfection of the adherent cells in 6-well plates, magnetic complexes were prepared by mixing 50 µl of a PEI-Mag2 MNP suspension containing 180 µg iron/ml water and 18 µl of the transfection reagent DF-Gold (OZ Biosciences, France). Then, 532 µl of the pDNA (pCD59-GFPBsd) solution containing 4.5 µg DNA in serum- and supplement-free DMEM was added to the mixture of particles and the enhancer, which resulted in 600 µl of the complex with a transfection reagent-to-pDNA ratio of 4:1 (v/w) and an iron-to-pDNA ratio of 2:1 (w/w). The mixture was further incubated at RT for 20 min to allow the components to assemble. After this time, 100 µl of the prepared complexes were added per well, with 75 × 10^3^ seeded cells in each well, resulting in an applied pDNA dose of 10 pg pDNA/cell. The mean hydrodynamic diameter (*D*_*h*_) and electrokinetic potential (*ζ*) of the transfection complexes in DMEM without additives were determined by photon correlation spectroscopy (PCS) using a Malvern Zetasizer Nano Series 3000 HS.

### Testing the DNA Binding Capacity of the Magnetic Nanoparticles

To evaluate pDNA association with magnetic nanoparticles in the presence of DF-Gold as an enhancer, aliquots (10 µl) of a 2:1 dilution series of the MNPs were added to wells in a 96-well round-bottomed plate starting from the concentration of 144 µg iron/ml. A 10-µl water sample was added to the reference well. Then, 12.96, 9.72, and 6.48 µl of the enhancer were mixed with 167.04, 170.28, and 173.52 µl of water, respectively. Afterwards, 20 µl of the enhancer solutions were added to appropriate wells containing 10 µl of MNP dilutions and thoroughly mixed. pDNA stock solution in DMEM medium without additives contained 2.4 µg/ml total ^125^I-labeled DNA with ^125^I (Hartmann Analytics, Germany) DNA-associated radioactivity of 2 × 10^5^ CPM/ml. Iodination of the DNA was performed in Pierce Pre-Coated Iodination Tubes, as described elsewhere [21]. Finally, 150 µl of the ^125^I-labeled DNA solution was added to each well and mixed, followed by incubation for 15 min to allow for complex formation. To sediment the magnetic transfection complexes, the 96-well plate was placed on a 96-well magnetic plate (OZ Biosciences) for 30 min. Afterwards, 50 µl of the supernatant was carefully transferred along with the pipette tip into the scintillation vial (Thermo Scientific, USA). Radioactivity was measured in each vial using a gamma counter device (Wallac 1480 Wizard 3, Perkin Elmer, Finland). Magnetically sedimented pDNA associated with the PEI-Mag2 nanoparticles was calculated as follows:

$${\text{Magnetically sedimented pDNA }} = {\text{CPM}}_{\text{ref}} - {\text{CPM}}_{\text{sample}},$$

where CPM_ref_ is the radioactivity measured in the reference well.

### Transfection of PFFs Using Magnetofection

For all transfection experiments, the PFF cells were seeded at a density of 9 × 10^3^ cells/cm^2^ in flat-bottomed plates pre-coated with 0.1 % gelatin solution. For transfection of the adherent cells in 6-well plates, 4 ml of the cell suspension (1.875 × 10^4^ cells/ml) was transferred into the wells, resulting in 7.5 × 10^4^ cells/well, providing a confluence of 50 % on the day of transfection 24 h after seeding the cells. One hundred microliters of the freshly prepared magnetic complexes were added per well resulting in an applied pDNA dose of 10 pg DNA/cell. The plate was positioned on the Mega Magnetic Plate (OZ Biosciences) and incubated for 20 min to sediment the magnetic complexes onto the cell surface. The cell culture plate was further incubated for 48 h.

### Intracellular Localization Study

To track the non-magnetic and magnetic complexes in the cell, the plasmid DNA (p55pCMV-IVS-luc+) was fluorescently labeled with fluorescein, according to the manufacturer’s protocol (Label IT^®^ Tracker™ Intracellular Nucleic Acid Localization Kits, Mirus Bio, USA). The final concentration of the fluorescein containing pDNA was quantified on a spectrophotometer (NanoDrop 2000, Thermo Scientific). The fluorescently labeled pDNA was then used to assemble non-magnetic and magnetic complexes, as described above. The intracellular localization of fluorescein labeled pDNA was monitored at 30 min, 4 h, and 24 h after the magnetic field was removed. For visualization under a confocal microscope, the cells were washed twice with Dulbecco’s phosphate-buffered saline (PBS; Sigma-Aldrich) and fixed with 4 % formaldehyde (Sigma-Aldrich) in PBS for 30 min. Afterwards, the fixative was removed and the cells were washed twice with PBS to dispose the formaldehyde. Cells on coverslips were mounted using VECTASHIELD anti-fade Mounting Medium containing DAPI (Vector Laboratories, USA) and imaged using a confocal laser scanning microscope (Olympus FV1000, Japan) equipped with a 405 and 488 nm laser for collection of DAPI and fluorescein emission signals, respectively. To detect nucleus and plasmid DNA inside the cells, two channels were used: blue (*λ*_ex_/*λ*_em_ = 373 nm/422 nm) and green (*λ*_ex_/*λ*_em_ = 488 nm/535 nm), respectively. The images were analyzed with FV10-ASW software (Olympus).

### Establishment of the Fibroblast Cell Lines that Stably Express the Transgene

After 48 h from magnetofection, the medium was replaced by culture medium supplemented with blasticidin selection factor (5 µg/ml) (Life Technologies, USA) and an 8-day selection was performed. At the end of the antibiotic-mediated selection process, the vitality of the cells was evaluated. The untransfected cells were used as a reference. The expression of eGFP (reporter marker) was verified by fluorescence microscopy. Selected, stable cell lines of PFFs were further cultivated until they reached full confluence in order to perform their molecular characterization on the isolated genomic DNA.

### Screening for the Presence of the Transgene in Generated Stable Cell Lines

The analysis of the presence of pCD59-GFPBsd transgene in the transfected cells involved genomic DNA isolation from porcine fetal fibroblasts using proteinase K (Qiagen, Germany), followed by amplification of two PCR fragments encompassing promoter–gene junction. Forward primers were complementary to the sequence of the EF-1α promoter and the reverse primers were complementary to the region encoding the CD59 factor. The PCR product of 333 bp was formed using F1 (5′-CTCGATTAGTTCTCGAGCTT-3′) and R1 (5′-AGCAGCAGCCCGAACAGGACAGAC-3′) primers, whereas the second fragment of 477 bp was amplified with F2 (5′-GGCCCTGCTGCAGGGAGCTC-3′) and R2 (5′-AGCAGCAGCCCGAACAGGACAGAC-3′) primers. PCR was performed in 20-µl reactions containing 100 ng of genomic DNA, 1× ReadyMix™ (Sigma-Aldrich) and 0.125 µM of each primer and the PCR amplification was conducted using the following conditions: denaturation 94 °C/30 s, annealing 57 °C/30 s, and synthesis 72 °C/45 s, 30 cycles. The PCR products were separated on a 1.5 % agarose gel (Sigma-Aldrich).

### mRNA Expression Analysis of Transgene

Expression of the CD59 cDNA in transfected cells was analyzed by reverse-transcription PCR (RT-PCR). Total RNA was isolated from porcine fetal fibroblasts using the Total RNA Mini Plus kit (A&A Biotechnology). The detection of human CD59 mRNA was carried out by reverse transcriptase reaction using the SuperScript^®^VILO™ cDNA Synthesis Kit (Invitrogen). For PCR, 1 μl of cDNA solution was used. PCR was performed using 35 cycles (primer annealing 56 °C; extension 72 °C; denaturation 94 °C). The 327-bp cDNA fragment was amplified using primer F (5′-CTCGTCCTGGCTGTCTTCTG-3′) and primer R (5′-TGCTGCCAGAAATGGAGTCA-3′). RNA purity control was performed by the same set of primers. Simultaneously, to check the quality of the cDNA, the 319-bp cDNA fragment was amplified using primer β-actin F (5′-ACATCAAGGAGAAGCTGTGCTAC-3′) and primer β-actin R (5′-CTTCATGATGGAGTTGAAGGTAGTT-3′). The PCR products were separated on a 1.5 % agarose gel.

### Chromosome Preparation

To arrest the cell division of porcine fibroblasts in mitotic metaphase, colcemid (0.05 µg/ml) (Sigma-Aldrich) was added to the culture dishes of 80 % confluence and the cells were incubated at 37 °C for 2 h. Then, the cells were harvested and suspended in a hypotonic solution (75 mM KCl) for 30 min to induce osmotic shock. The material was fixed with a cold mixture of methanol and acetic acid (3:1) three times. The number and dispersion of metaphase plates was evaluated during observation with a Nikon Eclipse E400 optical microscope in phase contrast mode. Karyotype evaluation involved staining of chromosomes with Giemsa stain solution. The obtained chromosome bands were analyzed using a microscope with a camera (Axio Imager M.2, Carl Zeiss Microscopy GmbH, Germany) and appropriate software (Ikaros, MetaSystems).

## Results

### Characteristics of Magnetic Nanoparticles Used for Nucleic Acid Delivery

**Fig. 2:**
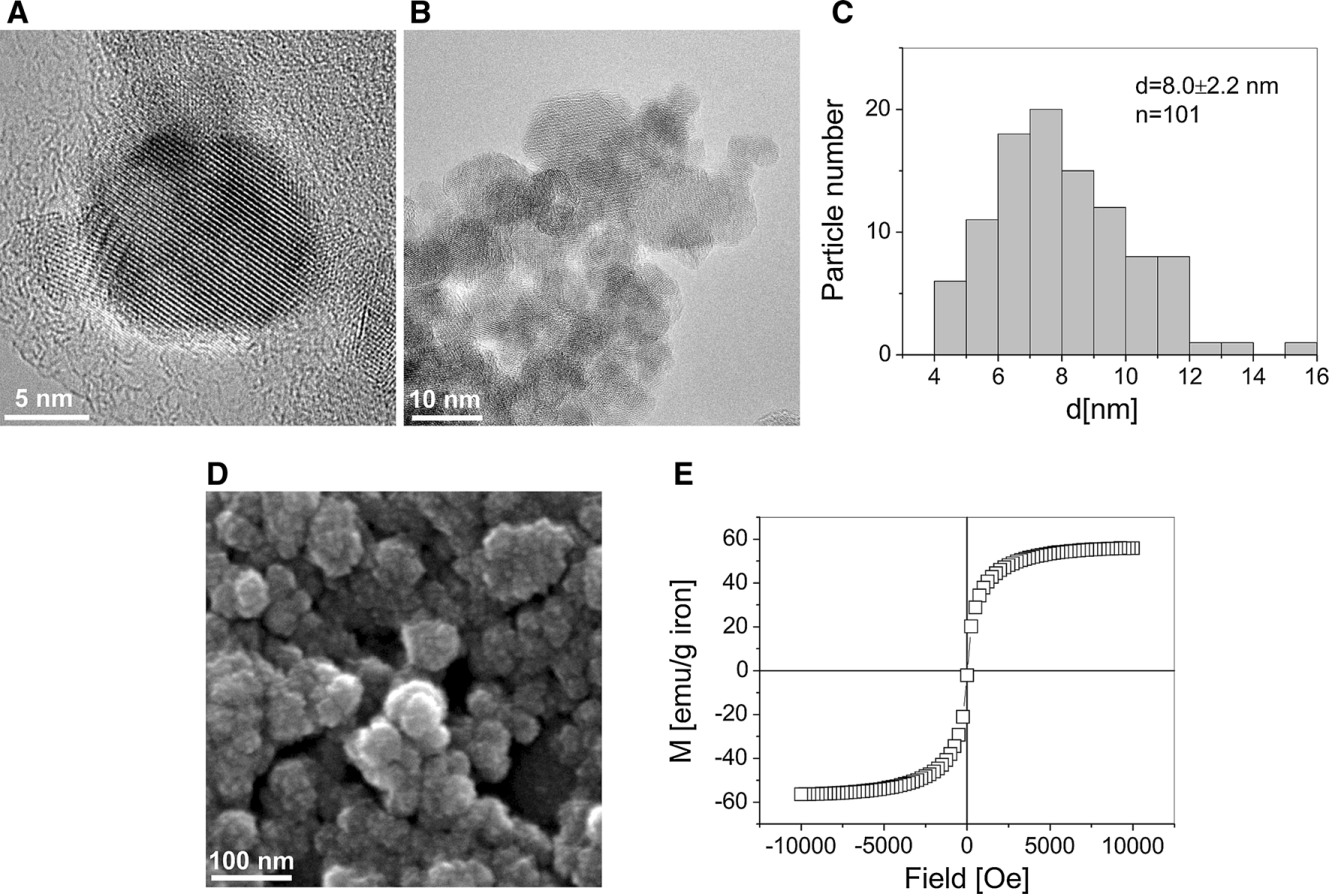
Characteristics of PEI-Mag2 magnetic nanoparticles used to assemble magnetic lipoplexes. **a**, **b** High-resolution transmission electron microscopy images. Spherical core shape and crystalline structure of the particles are clearly visible. **c** The size distribution of the particles based on quantitative analysis of the TEM images. **d** Scanning electron microscopy images demonstrating the spherical shape of the particles. **e** Magnetization curve displaying magnetic properties

PEI-Mag2 core–shell-type iron oxide magnetic nanoparticles (MNPs) with a surface coating suitable for nucleic acid delivery formulated from the fluorinated surfactant ZONYL FSA (lithium 3-[2-(perfluoroalkyl)ethylthio]propionate) and 25-kDa branched polyethylenimine (PEI-25Br) were used to associate magnetic transfection complexes. The size, morphology, ζ-potential, as well as magnetic properties of these particles were assessed. The particle sizes and morphology were evaluated by electron microscopy. The TEM micrographs show the presence of apparently spherical iron oxide cores (Fig. 2a, b) and quite uniform size distributions with the core size being of 8.0 ± 2.2 nm (Fig. 2c). The SEM image demonstrates the spherical shape as well as multicore structure of the MNPs (Fig. 2d). When drying, particles tend to agglomerate due to their high surface energy. Figure 2e presents the magnetic properties of PEI-Mag2 particles. The saturation magnetization registered at room temperature was 56 emu/g iron. The magnetization curve exhibited a superparamagnetic behavior due to zero coercivity and zero remanence. The mean hydrodynamic diameter measured in aqueous suspension was 28 nm, and the electrokinetic potential was highly positive at +48.8 ± 1.0 mV resulting from the presence of PEI in the surface layer of the particles.

### Testing Nucleic Acid Association with Magnetic Nanoparticles

**Fig. 3:**
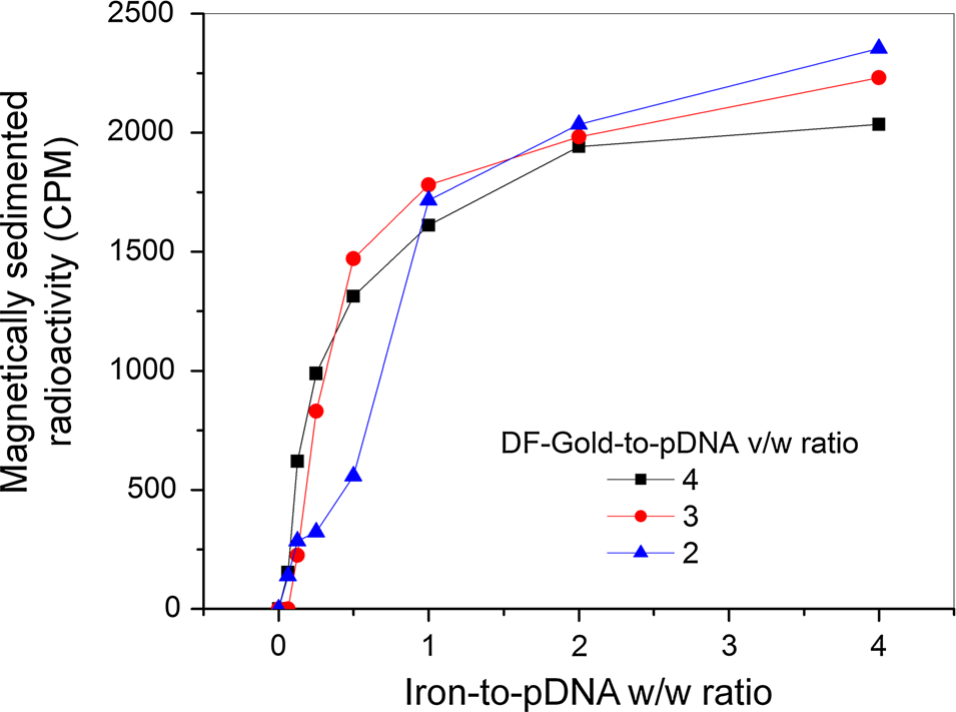
Magnetic sedimentation of pDNA associated with PEI-Mag2 nanoparticles in the presence of DF-Gold as an enhancer. pDNA associated and magnetically sedimented with PEI-Mag2 nanoparticles in magnetic lipoplexes after incubation at the magnetic plate for 30 min at different enhancer-to-pDNA v/w ratios (4, 3, 2) plotted against magnetic nanoparticle concentrations in terms of iron-to-pDNA w/w ratios (starting pDNA concentration of 2 µg/ml). A range of iron-to-pDNA w/w ratios from 0.0625 to 4 has been examined

To assess the pDNA binding capacity of the magnetic nanoparticles and the magnetic sedimentation of the resulting transfection complexes, the magnetic lipoplexes were formulated using ^125^I-labeled pDNA, DF-Gold as an enhancer, and PEI-Mag2 nanoparticles. A range of iron-to-pDNA ratios (w/w) from 0.0625 to 4 were tested. To sediment magnetic lipoplexes, the complexes were placed on the magnetic plate and after 20 min of incubation the radioactivity was measured in the supernatant. At all tested DF-Gold-to-pDNA (v/w) ratios from 2 to 4 and PEI-Mag2-to-pDNA iron w/w ratios higher than 1, the pDNA was to a significant extent associated and magnetically sedimented (Fig. 3), suggesting the potential usefulness of these complexes composition for magnetofection.

### Characteristics of the Magnetic Transfection Complexes

To measure the size distribution and electrokinetic potential of the magnetic transfection complexes, magnetic lipoplexes with DF-Gold and PEI-Mag2 MNPs at an enhancer-to-DNA ratio of 4:1 (v/w) and an iron-to-DNA ratio of 2:1 (w/w) were prepared. The average size of the complexes according to the hydrodynamic diameter was approximately 1800 nm. The complexes had a positive net charge with zeta-potential values of 8 mV when measured in DMEM medium.

### Cellular Uptake of Magnetic Transfection Complexes

**Fig. 4:**
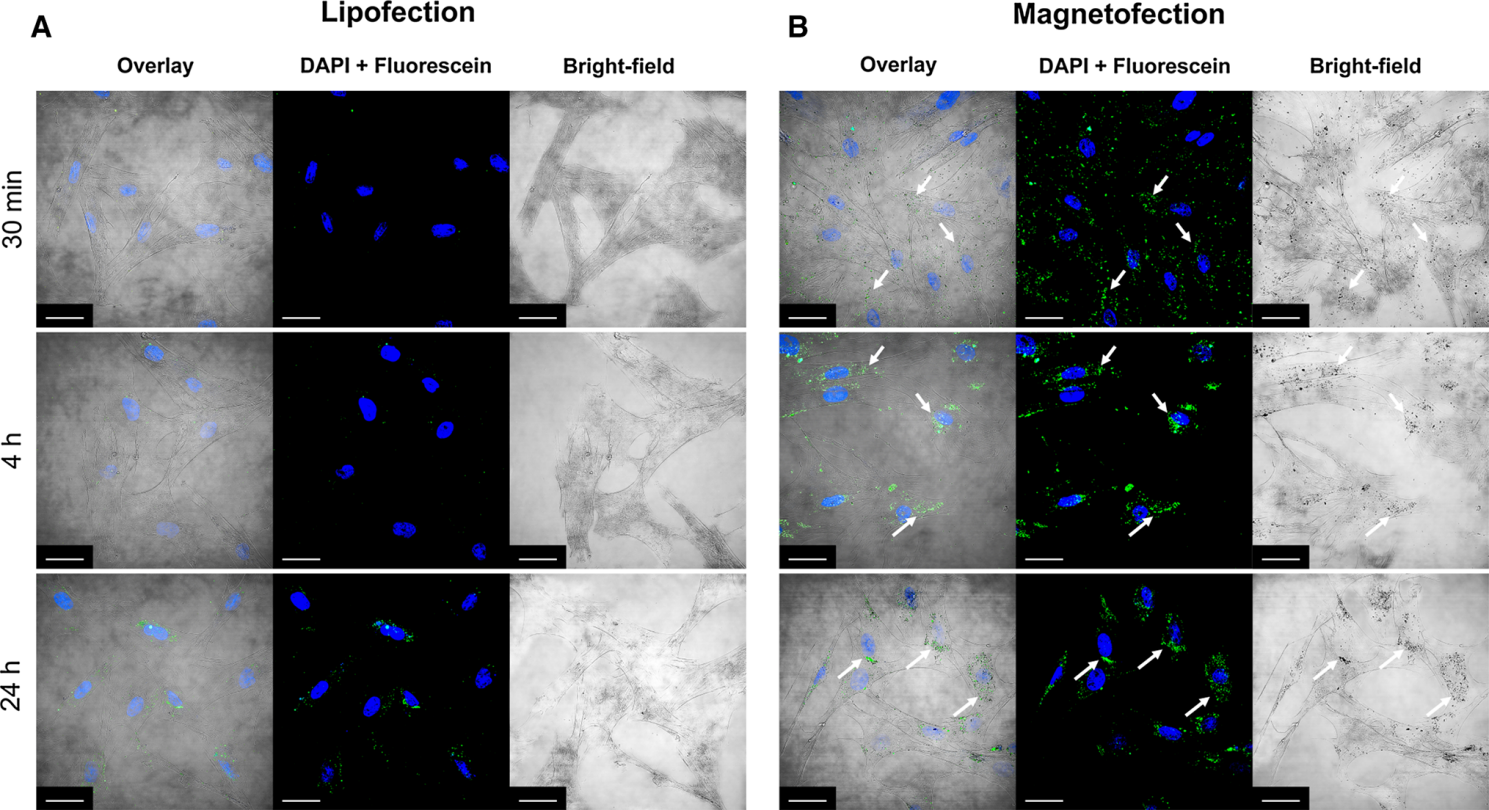
Intracellular localization of magnetic and non-magnetic transfection complexes in PFF cells. Fluorescein-labeled p55pCMV-IVS-luc+ DNA (*green*) was used to prepare non-magnetic (**a** lipofection) and magnetic complexes (**b** magnetofection) at 2:1:4 iron-to-pDNA-to-enhancer (w/w/v) ratio and added to the cells. Following 30 min, 4 h, and 24 h after incubation in a magnetic field, cells were fixed and stained with DAPI (*blue*), and visualized by confocal microscopy. *Arrows* indicate the presence of the magnetic lipoplexes in the intracellular compartment. *Scale bar* 25 µm (Color figure online)

**Fig. 5:**
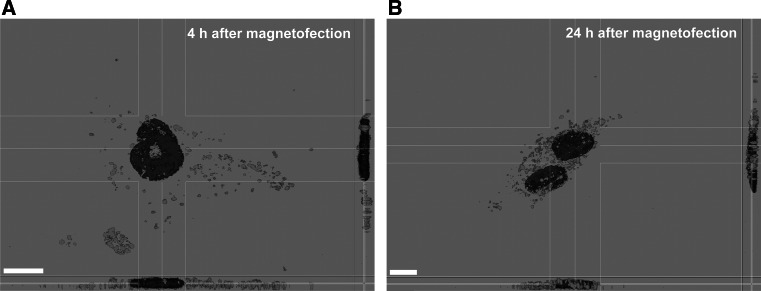
Interaction of the magnetic transfection complexes with PFF cells. 3D reconstruction of the *x*,*z* and *y*,*z* slices of the cells exposed to the magnetic transfection complexes containing fluorescently labeled pDNA (*green*) following 4 h (**a**) and 24 h (**b**) after incubation in a magnetic field. *Scale bar* 10 µm (Color figure online)

 In order to evaluate the intracellular localization of magnetic lipoplexes containing fluorescently labeled pDNA, magnetofection was conducted and fluorescent images were taken at 30 min, 4 h, and 24 h after magnetofection. The confocal microscopy confirmed rapid cellular uptake of the complexes during the magnetofection process. As shown in Fig. 4, enhanced uptake of the magnetic transfection complexes into the PFF cells was observed within 30 min after application of the magnetic field. At 4 and 24 h after magnetofection, fluorescent images show that the majority of the magnetic vectors were present around the nucleus (perinuclear localization) and inside the nucleus (Fig. 5 and Supplementary Video S1 and S2). In comparison, cells treated with non-magnetic lipoplexes showed significantly slower and lower uptake. Analysis of pDNA trafficking shows that magnetic vectors accumulate much faster than non-magnetic vectors in the cytoplasm as well as in the nucleus. The images indicate that upon application of the magnetic field, the full-applied magnetic vector dose is sedimented on the cells and internalized within a very short time period and, therefore, the vector dose requirement is considerably reduced. The optical images show the presence of black dots corresponding to the magnetic lipoplexes, which are not observed after lipofection.

### Generation of Modified Fibroblast Cell Lines

**Fig. 6:**
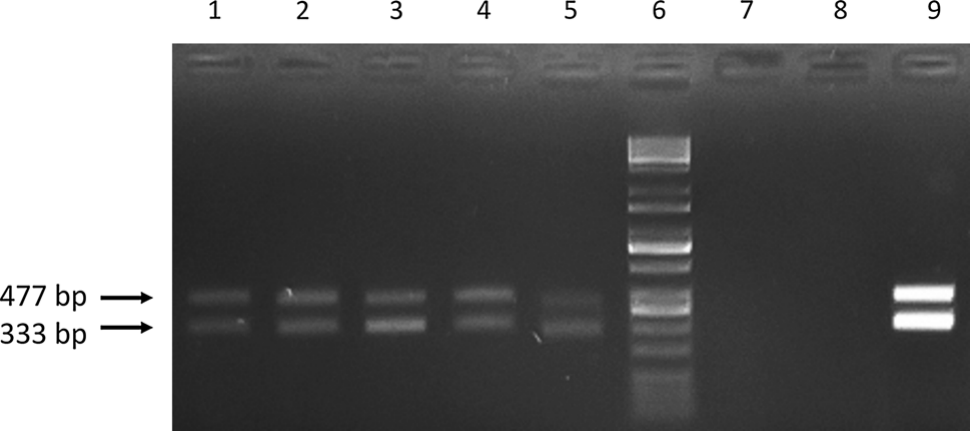
Screening of the pCD59-GFPBsd gene construct. PCR was performed to amplify DNA fragments of 333 and 477 bp. The PCR products were fractionated on 1.5 % agarose gel. Analysis of pCD59-GFPBsd integration with genomic DNA isolated from five transfected PFF cell lines. *Lanes 1–5* porcine fetal fibroblasts after magnetofection, *lane 6* size marker (Kapa Universal DNA Ladder), *lane 7* negative control (porcine DNA), *lane 8* negative control (without DNA), *lane 9* positive control (pCD59-GFPBsd gene construct)

**Fig. 7:**
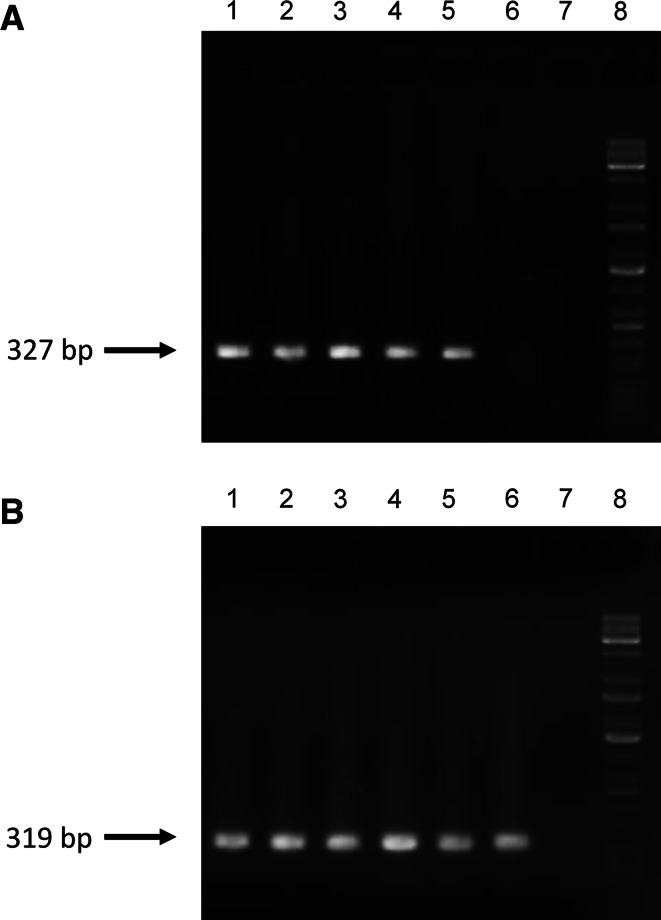
Detection of the CD59 mRNA expression in PFF cell lines after magnetofection. PCR amplification followed by agarose gel electrophoresis. **a** Analysis of pCD59-GFPBsd expression. **b** Analysis of β-actin expression (cDNA quality control). *Lanes 1–5* porcine fetal fibroblasts lines after magnetofection, *lane 6* negative control (porcine DNA), *lane 7* negative control (without DNA); *lane 8* size marker (Kapa Universal DNA Ladder)

The transgenic fibroblast cell lines were produced by magnetofection of porcine fetal fibroblasts with pCD59-GFPBsd gene construct containing the human *CD59* gene under the control of an hEF-1α promoter. After the selection process, fibroblast vitality and visual eGFP expression was evaluated. A screening procedure involving 333 and 477 bp PCR products was performed to identify transgenic fibroblast cell lines. After magnetofection, five individual cell lines (5/5) were found to be transgenic with the transgene incorporated into the nuclear genome. The results of screening for the pCD59-GFPBsd transgene are presented in Fig. 6. The results of RT-PCR showed the expression of human CD59 gene in transfected PFF cells (Fig. 7). Karyotype analysis of the transgenic cell lines performed with the Giemsa method revealed normal chromosome counts and structure in magnetofected PFFs (2*n* = 38).

## Discussion

The SCNT method enables transfer of most of the manipulations from a farm to a laboratory, where the modification of somatic cells is performed and the clones characterized for transgene integration into the genome are selected [22]. Efficient nucleic acid delivery into primary fibroblasts is a prerequisite for the successful generation of transgenic cells. Numerous methods of exogenous DNA introduction into porcine fibroblasts have been applied [8–10, 23, 24]. In our previous work [21], we showed efficient transfection of porcine fetal fibroblasts (PFFs) using magnetofection technology. Magnetic vectors formulated by the self-assembly of PEI-Mag2 iron oxide nanoparticles, plasmid DNA, and an enhancer in combination with an applied magnetic field enabled improved complex internalization and transgene expression, resulting in up to 60 % of transfected cells having high cell viability. This study describes the generation of stable transgenic cell lines, which can be used as donor cells for nuclear transfer with optimized magnetic transfection complexes, as shown schematically in Fig. 1. Plasmid DNA containing a coding sequence of human CD59 glycoprotein, which is an inhibitor of the membrane attack complex, was used to transfect PFFs. This protein is one of the components regulating the activity of the complement system mechanism responsible for the rejection of transplant organs. Its overexpression in transgenic porcine fibroblasts may ensure resistance to complement-mediated injury [25, 26].

The first essential step for efficient magnetofection is the formulation of nanomagnetic pDNA delivery vectors. For this purpose, we used PEI-Mag2 magnetic nanoparticles, which proved to be able to assemble magnetic transfection complexes and deliver different types of nucleic acid with high effectiveness [18, 20, 21, 27, 28]. In order to be useful in the magnetofection process, MNPs have to be specifically designed and engineered in terms of their chemical and magnetic properties. To create MNPs suitable for association with plasmid DNA through electrostatic interactions, the surface of these particles was modified with cationic polymeric 25-kDa branched PEI resulting in highly positive electrokinetic potential in water. This hydrophilic polymer is known to condense negatively charged nucleic acids, promote cellular uptake via endocytosis, and enable the endosomal escape of the complexes according to the “proton sponge” hypothesis [29]. The magnetic properties of the particles enable magnetic complex manipulation in the presence of the magnetic field resulting in rapid sedimentation of the full vector dose on the cell surface. The magnetization curve of the PEI-Mag2 particles is without hysteresis, thus demonstrating superparamagnetic properties. Particles exhibit such behavior when the core size is sufficiently small (below 15 nm) and they are well stabilized with an appropriate coating [30]. This is in agreement with the size of PEI-Mag2 particles determined by TEM. Despite the reduced magnetic properties (56 emu/g Fe) compared to bulk magnetite (127 emu/g Fe), PEI-Mag2 showed sufficient magnetization for efficient magnetofection. The decrease in the saturation magnetization of the nanoparticles compared to the bulk material can be related to the generation of the so-called magnetically “dead” layer on the surface and change of the magnetic ordering of the core due to surface functionalization of the nanoparticles with non-magnetic material dependent on the anchor functional groups [30, 31].

In the context of magnetofection, any agent that associates with MNPs and nucleic acid resulting in improvement of gene delivery can be used as an enhancer. Different parameters such as the choice of enhancer, optimal ratio of MNPs and enhancer to nucleic acid, and mixing order should be considered. The addition of cationic lipids or polymers as a pDNA condensation agent to associate complexes with MNPs led to the enhancement of MNP-mediated plasmid DNA delivery into various mammalian cells [32–34]. We have found that magnetic lipoplexes with transfection reagents DF-Gold combined with PEI-Mag2 MNPs facilitate vector internalization and endosomal escape as well as protect the nucleic acid against nuclease degradation yielding high exogenous gene expression. The analysis of the nucleic acid binding capacity demonstrated association of the pDNA and the PEI-Mag2 nanoparticles in the presence of DF-Gold. Data on the association and magnetic sedimentation of the magnetic lipoplexes plotted against MNP concentration indicated that almost complete magnetic sedimentation of the complexes under the experimental conditions occurred at an iron-to-DNA ratio higher than 0.5, suggesting the potential usefulness of these ratios for efficient gene delivery.

The size as well as electrokinetic potential of transfection complexes were found to be important factors affecting their effectiveness in cellular uptake. The DNA/(transferrin)-PEI complexes with an average size greater than 500 nm were more efficient in gene transfer compared to small complexes of 40 nm [35]. Ross et al. reported that the internalization of the DOTAP/DOPE lipoplexes in CHO cells increased with increasing lipoplex size and found the largest complexes (of 2.2 µm) to be the most efficient [36]. Li et al. showed that size, not surface charge is the major determinant of in vitro lipofection efficacy [37]. The mean size of the magnetic complexes formulated at an iron-to-DNA w/w ratio of 2 was 1.8 µm. These complexes possessed a positive surface charge of 8 mV in cell culture medium without serum. It has been reported that the net charge is involved in the binding of complexes to the cell membrane [38]. The electrokinetic potential of magnetic lipoplexes used in this study influences the efficacy of magnetofection through enhanced binding of complexes to the cell membrane resulting from non-specific ionic interaction between the positively charged magnetic lipoplexes with the negatively charged membrane.

The sufficient magnetic properties of the complexes enable accelerated sedimentation of the magnetic lipoplexes onto the cell surface upon application of the magnetic field. The magnetic field itself does not alter the mechanism of the magnetic complex uptake [39]. Exposure to a gradient magnetic field for 20 min was followed by intracellular localization of magnetic vectors containing fluorescently labeled pDNA 30 min, 4 h, and 24 h after magnetofection. We observed that for magnetic lipoplexes a majority of the complexes were attached to the cell membrane within 30 min after application of the magnetic field, and many of them were already visible in the cytoplasm. After 4 and 24 h, the presence of the pDNA was revealed in the perinuclear area as well as in the nucleus. Cellular uptake of labeled DNA showed that magnetic lipoplexes demonstrate better DNA delivery ability to the cells than non-magnetic lipoplexes. The advantages of magnetic field-mediated gene delivery can be attributed to enhanced concentrations of magnetic transfection complexes on the cell surface and reduced non-specific interactions with serum components from cell culture medium resulting in increased transfection efficiency [18]. In this manner, cellular internalization of the magnetic complexes is enhanced and the transgene expression is significantly improved.

Generation of a transgenic somatic cell line suitable for nuclear transfer requires stable transgene integration into the genome of each cell. Besides the gene of interest, the incorporated gene construct should also contain selection and visual markers. Only genetically transformed cells that survive selection can be used as a source of nuclear donor cells to reconstruct the enucleated oocytes. Various methods have been employed for the successful production of stable transformants [8, 10, 40]. In the present study, we established a porcine fibroblast cell line comprising the human CD59 factor gene under the control of the elongation factor (hEF-1α) promoter for the purpose of xenotransplantation. We used two selection markers, a blasticidin-resistance gene to isolate genetically modified cells and an *eGFP* gene to confirm the incorporation of the transgene with the genomic DNA. The magnetofection method which was applied to modify PFF cell lines proved to be an efficient and non-toxic strategy for producing transgenic nuclear donor cells. The magnetofected cell lines were characterized by high survival rates and proliferative activities. It was revealed that all five genotypically analyzed cell lines that had undergone the magnetofection were positive for pCD59-GFPBsd transgene screening. All of the transgenic cell lines were also eGFP-positive and expressed human CD59 gene. No morphological alterations were observed and there was a correct chromosome number and structure for magnetofected PFFs.

## Conclusions

This study demonstrates that magnetofection appears to be an alternative method for viral and non-viral gene delivery into porcine fibroblasts. Magnetic complexes formulated with a gene of interest under optimized conditions in combination with an inhomogeneous magnetic field enabled effective, rapid, and non-toxic transfection of PFFs, resulting in the generation of stable transgenic cell lines. Successfully established porcine fibroblast cell lines expressing human CD59 membrane proteins can be used as a nuclei source for SCNT. Further studies are needed to determine whether the use of a magnetofection method adapted for the purpose of SCNT can result in the creation of viable transgenic cloned animals for the purpose of xenotransplantation.

## Electronic supplementary material

Below is the link to the electronic supplementary material.

Supplementary material 1 (MP4 8426 kb)

Supplementary material 2 (MP4 8424 kb)
